# Tailored Psychoeducational Home Interventions for Children with a Chronic Illness: Families’ Experiences

**DOI:** 10.5334/cie.100

**Published:** 2024-01-05

**Authors:** Michele Capurso, Gaetano Catalano, Anna Calvaruso, Annalinda Monticelli, Calogero Taormina, Samanta Battiato, Francesca Paola Guadagna, Tania Piccione, Paolo D’Angelo, Delia Russo, Antonino Trizzino, Veronica Raspa

**Affiliations:** 1 Department of Philosophy, Human and Social Sciences and Education, University of Perugia, IT; 2 SAMOT ONLUS, PA, IT; 3 Social Policies Lab Foundation –Labos E.T.S., IT; 4 Department of Pediatric Hematology Oncology A.R.N.A.S. Civico, Palermo, IT; 5 SPIA ONLUS, IT; 6 ASLTI ONLUS, IT; 7 SAMOT ONLUS, IT; 8 Research assistant, Department of Philosophy, Human and Social Sciences and Education, University of Perugia, IT

**Keywords:** Development, children and young people with a chronic illness, home intervention, family, parents, resilience, support

## Abstract

The quality of life for a child with a chronic illness depends on various factors, including the illness’s severity, medical treatments, psychosocial and educational support, resource availability, and community involvement. These biopsychosocial factors become significant when the child receives care at home. This article presents and evaluates a highly personalized support project offered to 40 Sicilian families, consisting of educational, social, and psychological services delivered at the families homes and in their communities. Guided by the Psychosocial Assessment Tool (PAT) and the Functional Psychology framework, the project employed a family-focused approach to healthcare and was based on a continuous dialogue between all stakeholders. The project was evaluated through a qualitative interview with eight families in the Palermo area, which was analyzed using consensual qualitative research. Results revealed families’ appreciation of the project and the importance of a professional who listened to their needs, provided a connection with the medical team, and tailored activities inside and outside the home. The ability of professionals to listen and adapt activities to different contexts and needs was crucial for the project’s success. We conclude that creating tailored family-level interventions with an educator acting as a liaison with the medical team is a widely acceptable strategy that should be further developed and investigated.

Approximately one in two hundred children in Italy have one or more chronic illnesses (Ministero della Salute, 2016). The care of these children requires close coordination between hospital and community care providers and ongoing interactions between professionals, parents, and the community (Galligan & Hogan, 2021). The quality of life for the child and their family is influenced by many factors such as the type and severity of the disease, psychosocial and educational support within the family and the local community, access to medical and other services, effectiveness of treatment and rehabilitation, and community acceptance and connectedness (Marchetti et al., 1995; Mitchell et al., 2020; Yang et al., 2022).

Illnesses and their treatments can cause significant psychosocial stressors and side effects that can negatively impact the quality of life of both the child and their family; additionally, they may hinder the child’s ability to participate in common social and educational activities that are otherwise important for healthy development, such as play, sports, and school (Ospina et al., 2021). However, psychoeducational interventions and physical and psychological therapies designed to support children and their families in managing the psychosocial consequences of chronic diseases and their treatments can alleviate such negative side effects (Barlow & Ellard, 2004; Yang et al., 2022).

Medical advancements have improved the survival rate of children with severe illnesses, and hospital stays have generally become shorter (Howlader et al., 2021; Price et al., 2012). Children with chronic medical conditions at home are challenged with readapting to various physical, emotional, and routine changes (Mitchell et al., 2020). For example, hospitalizations and health issues often compromise school attendance, affecting their socialization with peers (Nap-Van Der Vlist et al., 2020). Therapies may also cause changes in their physical appearance that can worry many children. These factors combined can have negative psychological effects on the developing child (Askins & Moore, 2008). For all these reasons, delivering psychoeducational intervention directly in the child’s own home is important (Galligan & Hogan, 2021).

Families face similar challenges and adjustments when dealing with chronic illnesses and similarly need emotional and practical support (Collins et al., 2020). Homecare involves practical and sometimes financial reorganization of family life. It requires a new balance between a sense of “normality” and the need to cope with the many demands posed by the illness (Van Schoors et al., 2018).

Given the above challenges, focusing on their sense of belonging is key to supporting children with chronic medical conditions. This focus is achieved by empowering them to interact socially and keep up with their peers (Hung & Chen, 2008; Tomberli & Ciucci, 2021). Based on these premises, the Italian Ministry of Work and Social Policies established a special fund in 2017 to support children with oncology diseases and their families (Law No. 205 of December 27th, 2017). Following the establishment of this fund, several charities and not-for-profit organizations developed projects to improve the quality of life of children and their families in different areas of the country. This study reports on the progress of a project run by SAMOT (Società Assistenza Malati Oncologici Terminali), a palliative and social care association in Palermo (Italy). It reports the qualitative results as perceived by a group of parents.

## Methods

The intervention described in this study consists of several methodological stages, including the initial Learning by Play: Growing Together project, a specific method for assessing family functioning, and specific methodologies for planning, delivering, and personalizing the at-home psychoeducational intervention. These stages will be explained in detail below.

### The “Learning by Play: Growing Together” Project

The Learning by Play (LbP) project was initiated in 2021 by the SAMOT association in collaboration with the Libertas association (both in Palermo, Italy) and the Foundation for Social Policy Studies (Labos) located in Rome, along with two children’s oncology hospitals in Sicily. The project combined social support principles with “exercise oncology,” which aims to improve patients’ health through regular, highly customized structured exercises (Adams et al., 2021; Watson et al., 2022). Additionally, sports and exercise programs provide opportunities for children to engage in positive peer interactions and learn new skills. These opportunities help families overcome social isolation and the stigma associated with cancer and other chronic diseases (Schmitz, 2020).

The main goal of the LbP project was to help children with chronic illnesses feel included in society and to sustain family development. This goal was achieved via two types of activities: (a) Play, sport, and leisure activities to children with medical conditions, following the principles of exercise oncology and with the guidance of a medical and rehabilitation expert; and (b) psychosocial, emotional, and relational support to families, young patients, and siblings.

Specifically, the objectives of the LbP project were: (a) Promote the biopsychosocial well-being of children with a chronic medical condition; (b) Provide access to free and tailored psychosocial activities in combination with the therapeutic path to support children holistically in their lives and development at times of illness; (c) Facilitate the participation and inclusion of children in school activities even when they must stay home or in the hospital due to their condition; and (d) Use technological solutions to support children’s relationships and sense of belonging through play- and sport-based social activities.

The LbP project started December 1, 2021, and ended in February 2023. During this period, it encompassed 40 Sicilian families, including 73 children and young people with a medical condition (mostly hematological and/or oncological) and some of their siblings. The project activities were managed by 20 professionals (i.e., psychologists, educators, social workers, administrators, and child psychomotrists) under the coordination of the SAMOT association’s project leaders.

One characteristic of the project was participation fluidity, which was applied at both the staff and family level. Staff participation was fluid because staff members were involved with families to deliver various services throughout the project. However, the hospital psychologists ensured the continuity of care throughout the project by acting as liaisons between the oncology-hematology departments and the families and supervised all the home interventions. Family participation in the project was flexible and subject to factors such as medical, biopsychosocial, and contextual conditions that changed over time. Some children had to leave the project due to declining physical health, while others could join later after receiving medical clearance. Additionally, the COVID-19 pandemic, which began in March 2020 and resulted in strict lockdown measures, continued to cause concerns for vulnerable individuals until at least the summer of 2022.

**Figure 1:**
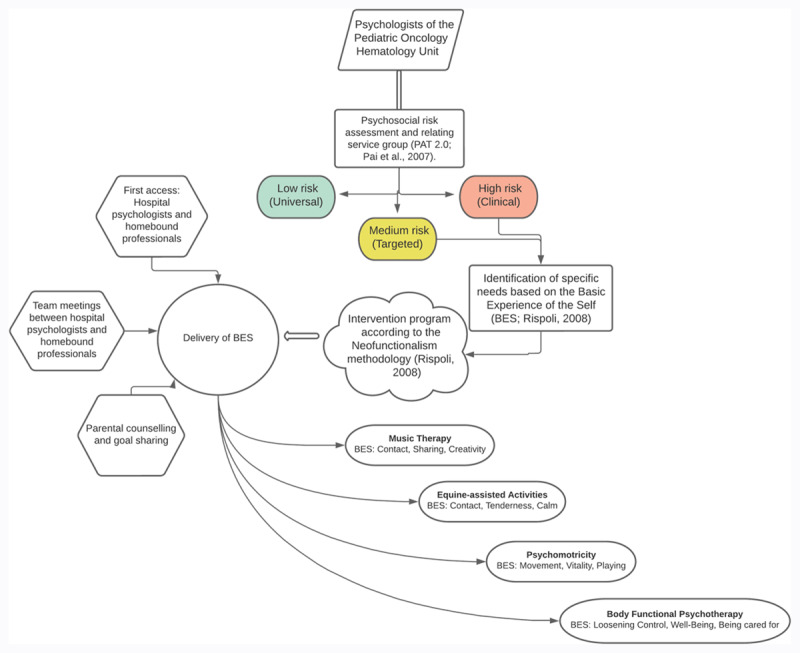
Personalization Process Used by the Team of Psychologists, Department of Pediatric Hematology Oncology A.R.N.A.S. Civico, Palermo.

The assessment and related personalization of the psychoeducation homebound intervention plan was based on a series of steps designed to meet patient and family needs, utilizing the competencies of the project team and contexts while simultaneously creating connections with community resources. We describe the model implemented by one of the project partners to explain the different steps of the personalization process, namely, the psychologists of the pediatric hematology-oncology department at the Hospital Arnas Civico di Cristina Benfratelli’s multidisciplinary team in Palermo, which served 13 families who participated in the project. The t steps of the assessment and consequent planning of the personalized interventions are shown in Figure 1.

### Assessment of Family Functioning

The project used the Pediatric Psychosocial Preventative Health Model (PPPHM; Kazak, 2006) a framework for understanding the impact of illness on families. The model highlights the importance of considering the social environment when assessing the child’s and family’s strengths and vulnerabilities (Kazak & Noll, 2015). To assess family needs, the PPPHM utilizes the parent-report screener Psychosocial Assessment Tool (PAT 2.0; Pai et al., 2007), which classifies families into Universal, Targeted, or Clinical categories based on psychological risk and subsequently recommends appropriate services. The PAT has strong psychometric properties and effectively identifies child behavior and parent stress (Pai et al., 2007). A corresponding set of support activities was developed for each category, varying in sensitivity and clinical characteristics.

### Personalization of the Intervention

The literature suggests that providing intervention components without considering the child’s and family’s specific needs is not productive (Mitchell et al., 2021). It is important to focus on the child’s and family’s key characteristics to identify the most effective and well-organized intervention options. The LbP project implemented personalized interventions based on assessing each family’s risk level. These interventions were designed to fit the individual needs of the patients and their families, considering the available local care systems (Galligan & Hogan, 2021).

To guide personalized intervention planning, the LbP project used an approach based on the functional psychology theory, which focuses on body image and meaningful physical experiences within the environment (Rispoli, 2008). Specifically, according to functional psychology, every experience involves a holistic understanding of the person as an integrated organism with various functions and subsystems (central and peripheral nervous systems, psychological and endocrine subsystems, physical sensations, emotional states, etc.).

Each holistic intervention can promote the development of resources to manage stress, cultivate well-being, and maintain good mental health by simultaneously addressing different systems in different ways (Rocco et al., 2022). For example, an equine-assisted activity impacts the physiological and postural system through horseback riding techniques, the emotional system through awareness of touch, the relational system through interactions with the animal and others, and the emotional-cognitive system using images and words to recall and share personal memories. The functional psychology theory identifies a set of Basic Experiences of the Self (BES; Rispoli, 2008; see Supplementary File 1) as a tool. These experiences have cognitive, emotional, physiological, and postural effects throughout life. Consequently, individuals gradually develop stable capacities that help them navigate and effectively manage various life situations.

In summary, the initial PAT assessment determined the risk level for each family. Based on the results, psychologists identified a set of missing or misconfigured BES, which, in turn, formed the foundation for the intervention. The most identified BES were contact, vitality, sharing, and tenderness. Table 1 provides an overview of the interventions implemented during the project.

**Table 1:** Description of the Main Activities of the LbP Project Delivered to 14 Families in the Palermo Area.


ACTIVITY NAME	DELIVERED BY	SUITABLE FOR PAT CATEGORY SERVICE GROUP	DESCRIPTION OF THE ACTIVITY	CORRESPONDING BES	TOTAL HOURS DELIVERED

Music therapy	Psychologist with music therapy certification	Targeted, Clinical	Work on emotions and relationships to build trusting relationships using nonverbal communication (communicating through the language of sound).	Losing Control, Joy, Vitality, Sharing	72

Psychosocial support to the family	Psychologist	Targeted, Clinical	Provide counseling to help the family deal with specific problems connected to the child’s education, illness, or other difficulties identified at the family level.	Being Held, Contact, Receiving Attention, Being Carried	247

Psychosocial support to the child	Psychologist	Targeted, Clinical	Provide counseling to help the child deal with specific tasks or behaviors connected to their illness or therapy.	Being Held, Being Carried, Control, Contact	270

Support with school	Educator	Targeted, Clinical	Provide support with homework and specific metacognitive functions such as attention, planning, problem-solving, studying, and memory.	Soft Attention, Autonomy Receiving Attention	120

Psychomotricity	Psychomotrist	Targeted, Clinical	Support motor and coordination abilities while also facing relational difficulties associated with the disease, such as shyness. Promote open and positive physical postures through playful motor activity.	Vitality, Control, Sharing	60

Equine-assisted activities	Educator	Targeted	Arrange activities that involve interacting with horses to address physical, mental, and social issues. These activities are designed to foster growth and development.	Active Contact, Sharing, Tenderness	120

Cognitive stimulation intervention	Psychologist	Targeted	Harmonize attention through commitment and respect for the rules and establishing positive relationships.	Control, Sharing, Being Carried	80

Home-based play-educational physical activity	Physical Education Teacher	Targeted, Clinical	Arrange physical activity with a playful educational value to re-establish a harmonious relationship with the child’s body.	Vitality, Control, Sharing	30

Dance-movement activity	Psychomotrist	Targeted, Clinical	Explore the possibility of more open, freer movement, to overcome postural and character shutdowns related to hospitalization, and to unload the negativity linked to the disease experience.	Vitality, Calm, Sharing	71


*Note*: Families PP5 and PP10 were omitted because they withdrew from the project.

In line with the PPPHM, the specific schedule and delivery method of the intervention varied for each participant. However, all activities were grounded in a common set of psychoeducational principles as follows:

1. Employ a family approach based on a constant dialogue among the healthcare professional intervening at home, the parents, and the children or young people involved.
2. Make the home the focal point. Numerous activities were conducted at home because it was considered the central location for the family, providing a well-known environment where members could be themselves and where the family was in charge of welcoming the healthcare professional (and not the other way around).
3. Value play and recreational activities as an arena where the child can experience success and good individual functioning.
4. Establish a working environment based on mutual collaboration and open communication between home educators and hospital-based healthcare professionals.

## Evaluation of the Intervention

Given the complexity, fluidity, and diversity of the children, families, and professionals involved in the LbP program, we selected a qualitative evaluation to report on the way the families perceived the project. The evaluation was based on semi-structured qualitative interviews (Gianturco, 2020), designed and carried out to allow parents to express their family-centered point of view on the project.

### Participants

A subset of families who participated in the LbP project was invited to participate in the present study via an interview to enable us to report on the project activities coherently. The selection was based on the following criteria: (a) The family consented to participate in the different data-gathering phases, the recording of interviews, and its use to generate this report; (b) The family consistently participated in the proposed activities throughout the project; (c) The activities were carried out in the city area of Palermo, where the evaluation took place.

Initially, the project targeted 16 families in the Palermo area. Two families withdrew from the project in its initial phase. Out of the remaining 14, eight consented to be interviewed and reported. The characteristics of the child’s illness, interviewee demographics, and home- and family-based interventions are reported in Table 2.

**Table 2:** Overview of the Eight Families Interviewed, the Child’s Condition, and Related Home-Based Interventions.


CODE	INTERVIEWEE (AGE)	CHILD CONDITION	CHILD AGE	PAT CATEGORY SERVICE GROUP	TOTAL HOURS OF DELIVERED ACTIVITIES, CONTEXT	TYPE OF DELIVERED ACTIVITIES	MAIN ACTIVITY AIMS	ACTIVITY OBJECTIVES

PP4	Mother (42)	ALL	6	Targeted	36, H	Music therapy	Provide psychological home support to child and parents	Facilitating soft motor movements; helping the child to concentrate and follow family rules; support of parenting skills.

					36, S	Interven-tions at school	Promotion of basic learning	Growth through games, drawings, and playful-creative activities to develop basic skills and to enhance the relational aspect of “doing together.”

					36, O	Equine-assisted activities	Improvement of motor coordination through a relationship with nature	Improvement of relational difficulties related to the illness, to improve posture and closed attitudes through playful motor activity.

PP6	Mother (57)	MDB	17	Clinical	20 H	Psycho-logical support to the child	Intervention in compliance and symptom management	Improving the locomotor system through new action possibilities.

PP8	Mother (53)	CVID CRMO	18	Clinical	20 H	Psycho-logical support to the child	Intervention in compliance and symptom management	Construction of therapeutic alliance and interventions for symptoms such as sleeping difficulties, increased psoriasis, and worsening of general well-being. Interventions to support the well-being and university choice of the patient, who is conditioned by fear.

					20 H	Psycho-logical support to the family	Supporting parenting skills	Helping parents to understand and meet the autonomy needs of their child.

PP9	Mother (45)	SCID	6	Clinical	36 H	Psycho-social support to the child	Intervention in compliance and symptom management	Support connected to communicating about a medical relapse.

					36 H	Psycho-logical support to the family	Psychological support to the family and advice on the Family Protection Project	Increasing awareness of the child’s resources despite the limitations imposed by the illness.

					37 H	Support of school learning	Support of basic learning and social skills	Growth through games, drawings, and playful-creative activities to develop basic skills and to enhance the relational aspect of “doing together.”

PP11	Mother (36)	ALL	13	Clinical	50	Psycho-logical support to the child	Supporting and developing an alliance and symptom management	Improving the child’s locomotor system through new actions.

					20	Psycho-logical support to the family	Supporting and listening to parents’ difficulties in managing their children’s illness	Improving the ability to manage relationships and communicate with the child.

PP13	Mother (53)	PHP, DI, CNS-GCT	17	Targeted	90 H	Music therapy and cognitive rehabili-tation	Tackling some functional and relational difficulties related to the disease experience in a playful way	Promotion of social aspects in response to the isolation caused by the physical illness. Support of cognitive functions and development of personal interests related to music.

					30, O	Equine-assisted activities	Improvement of motor coordination through a relationship with nature	Improvement of relational difficulties related to the illness, to improve posture and closed attitudes through playful motor activity.

					100 H	Dance-movement activity, play-educational physical activity	Improvement of motor difficulties, coordination	Exploring the possibility of a more open, freer movement to overcome postural and character closures linked to hospitalization and to release the negativity linked to their experience of their illness.

PP14	Father (65)	CNS-GCT, DI, PHP	18	Targeted	10	Psycho-logical support to the child	Establishing good compliance and promoting health management autonomy	Supporting the patient to increase their self-awareness, understanding growth, and recognizing personal resources to manage social and family challenges.

					20 H	Psycho-educational support to the family	Supporting the family to manage the onset of Alzheimer’s in the mother	Reducing the family’s isolation and offering support to manage the mother’s behavioral issues. Addressing and preventing family distress caused by the mother’s illness while providing support and education on Alzheimer’s management. Creating an environment where the son can prioritize his studies and well-being, allowing him to step back from the overwhelming responsibility of caring for his mother.

PP15	Mother (46)	LCH	12	Targeted	98	Psycho-logical support to the family	Providing support for parenting skills and managing issues related to parenting difficulties	Enhancing family well-being and helping to manage quality time together. Special consideration given to the patient’s emotions, sensations, desires, and fears, as well as those of the single mother. Granting the mother additional leisure time to attend to her other child and her own needs. Alleviating chronic family stress associated with the patient’s condition.

					91	Psycho-educational and play-based support to the child	Improving emotion regulation and peer interaction	Recognizing and regulating emotions to develop greater self-awareness and awareness of others. Preparing the child for re-entering school after the COVID-19 lockdown and facilitating positive interactions with their peers.

					10	Equine-assisted activities	Improvement of motor coordination through a relationship with nature	Promoting awareness of own body posture and improving personal well-being through nature.

					20 H	Dance-movement activity	Promoting greater awareness of one’s body concerning emotions	Promoting an awareness of own body through play activities. Providing the means to express and share emotions. Improving motor autonomy.


*Note*: **Medical conditions:** ALL = acute lymphocytic leukemia. CNS-GCT = central nervous system germ cell tumor. CRMO = chronic recurrent multifocal osteomyelitis. CVID = common variable immunodeficiency. DI = diabetes insipidus. HL = Hodgkin lymphoma. LCH = Langerhans cell histiocytosis. MDB = medulloblastoma. PHP = panhypopituitarism. SCID = severe combined immunodeficiency. Ts21 = Trisomy 21. **Activity settings:** H = Home. I = individual. G = group. O = outdoor or community center. S = School.

### Interview Method

The open-ended interviews took place from January-April 2023, following all the ethical considerations and approvals set by the funding institution of the LbP project and following the participant’s consent. Interviews were held online and recorded while the parents were at home. They were conducted by one of the authors (AM), who holds a PhD in the sociology of cultural processes and has relevant experience in qualitative and mixed methods research. Sessions lasted 31–49 minutes.

All interviews began by AM requesting the parents to narrate their experiences with the LbP project. Examples of the questions are, “Please tell me how you experienced this project” and “What activities are the project’s professional undertaking with your child?” Further questions, such as “Can you explain what you mean by …” and “How did you feel when …,” were used to probe for greater depth. Additional follow-up questions were posed to clarify statements and to confirm the interviewer’s correct understanding.

### Data Analysis

The data analysis followed the established guidelines for consensual qualitative research (Hill et al., 2005). This methodology provides a rigorous systematic qualitative research method to investigate the subjective experience of a few participants to create rich descriptions of the experiences.

Data were analyzed through a consensual process involving three researchers and three phases: the creation of general domains reflecting general content, the creation of related summaries of texts, and the creation of categories based on common “core ideas.” Subsequently, frequencies were calculated to determine how the individual categories represented the entire sample and labeled using conventional terms. The researchers conducting the analysis were an associate professor of developmental psychology with expertise in educating children and young people with medical needs, a psychology researcher with relevant experience in consensual qualitative analysis, and a sociology researcher with relevant experience in qualitative research.

### Coding Process

First, the researchers read all the transcripts of the interviews and identified the various subject areas. Subsequently, the researchers discussed each interview’s segment and assigned it to a core idea until a final list of common core ideas was created. Next, the researchers developed similar core ideas for a more general category to summarize all similar core ideas. Once the list of categories was completed, the researchers reviewed the transcript material to determine whether each text segment was placed in the appropriate category group. Finally, the researchers placed coherent categories in a more general domain by consensus. In line with the recommendations of Hill and colleagues (2005), when a category occurred in more than half of the participants (in our case, more than five interviewees), we termed it “typical” and reported it as “most participants” who responded in a particular way. Core ideas cited by fewer than half the participants were termed “some” and identified as “some participants” who responded in a particular way. Low-frequency topics (two occurrences) were labeled “variable,” and very rare occurrences (1–2) were labeled “rare.”

## Results

Table 3 shows an overview of categories and the eight domains identified.

**Table 3:** Results of the Parents’ Interviews Regarding Their Perceptions of the Effects of the LbP Project (N = 8).


DOMAINS AND CATEGORIES	FREQUENCY*

**I. Health status of the patient during the project**	

**A.** The patient participated in the project’s activities during their illness (8/8).	General

**B.** The patient participated in the activities after recovering from the illness, but they were still experiencing psychological after-effects (5/8).	Typical

**II. Invitation and motivation to participate in the project**	

**A.** The patient’s mother or doctor proposed participation in the project to provide support, help the child assimilate the disease process, promote socialization, and enhance the physical component (8/8).	General

**III. Description and delivery of the LbP activities and attendance**	

**A.** The objectives were to support the development of improved self-image, socialization, and autonomy (8/8).	General

**B.** The participation of patients in the project varied in different contexts, with sessions offered several times a week and with different combinations of activities (i.e., physiotherapy, music therapy, play therapy, homework, sewing, communication, school psychological assistance, and gardening) (4/8).	Variable

**C.** The project activities were free of charge for families and conducted at home and elsewhere, as appropriate. The activities were chosen based on the patient’s needs and attitudes (6/8).	Typical

**IV. Barriers and negative aspects of the project**	

**A.** The restrictions imposed by the emergency health situation due to COVID-19 and the resulting parental concerns hindered the smooth progress of the project activities (5/8).	Typical

**B.** The project was ineffective in getting to know the patients and keeping them involved over time (2/8).	Rare

**V. Positive effects of the project on children and families**	

**A.** The project had positive effects, helping users to feel happy, calm, and protected. It also assisted them in their personal growth, socializing, coping with COVID-19, and enhancing their autonomy (8/8).	General

**B.** The positive effects of the project included the support of families and collaboration with professional figures and hospitals (7/8).	General

**VI. Perceived patients’ feelings associated with activities**	

**A.** Patients recalled the project activities and professionals positively (6/8).	Typical

**B.** Patients were tired, anxious, or angry towards people or activities connected with the project (5/8).	Typical

**VII. Future development perspectives**	

**A.** The project should be repeated with the following changes: support parents more, reinforce planning, have prevention and COVID-19 emergency protocols, provide more opportunities for sharing with the other participants in the network, intensify activities, and include siblings (6/8).	Typical

**VIII. Hospital as a negative place**	

**A.** The hospital is mentioned as a place of illness, stressful events, and restrictions (3/8).	Variable


*Note*: Total *N* = 8. *General: 8–7 occurrences; typical: 6–5 occurrences; variable: 4–3 occurrences; rare: 2–1 occurrences.

### Domain I

Domain I deals with the description of the participants’ health. Here, the parents described the different physical pathologies of their children (e.g., a rare disease of the immune system, leukemia) and provided an overview of their child’s functioning at the beginning of the project and throughout. See Table 3, section A of Domain I (I/A), with eight occurrences out of eight participants (8/8). For example, one mother said:

*“My daughter is immunocompromised and suffers from epileptic seizures, so she often stays at home, and it benefits her when the LbP’s operators come to be with her because, like all girls her age, she also has desires and dreams that the project helps to realize.”* (PP13)

The second typical category (I/B, 5/8 occurrences) describes patients who participated in the LbP project after recovering from an illness but were still dealing with comorbidities or psychological after-effects. Sometimes, the parent described the patient using terms reflecting severe psychological issues such as anxiety, depression, and so on. According to one mother:

*“My son has recovered from the organic illness, but he has some side-effects that still need to be overcome. So, rightfully, he still has to come to terms with what he has faced day by day.”* (PP6)

Parents also mentioned the importance of interventions beyond the traditional boundaries, such as professionals going to the patient’s home to act within the context of the whole family:

*“My daughter’s medical condition often prevents her from going out, so caregivers come to our home to be with her, and she gets along very well with these professionals. In fact, she can’t wait to see them and asks me every time, “Mom, when are they coming?”*

*Even we, her family, have established a good relationship with the professionals who don’t just focus on the patient. Together, we all become a family because, as a mother, they provide support when my husband is not around. They help me and explain the problems*.

*After the COVID lockdown, the caregivers organized outdoor events such as visits to the botanical garden and various field trips. It was important for my daughter, who has some challenges, as she could socialize in small, protected groups. For instance, my daughter was thrilled about the trip with the animals and the boat ride*.

*As parents, we would have never taken her on these experiences because of our fears, but the caregivers encouraged us. Just seeing our children happy is enough to make me feel more at ease.”* (PP13)

### Domain II

Domain II and its related single category (II/A, 8/8) deal with access to the project and its main perceived motivations, described as providing support, helping the user process the disease, providing socialization opportunities, and enhancing physical activity.

*“The doctor we met at the hospital proposed to my daughter to participate in the LbP project to socialize with other kids, talk about her illness, and perhaps help her understand that there is more to life than just her illness*.

*My daughter refuses to go to the hospital because she sees the illness and typical problems of that environment, so she doesn’t socialize much there. That’s why the doctor tried to involve my daughter in the project, to have her interact more with other people and help her understand that it is possible to face the illness with the help of others.”* (PP8)

### Domain III

Domain III describes the LbP activities and includes a general category, III/A (8/8), about the objectives to support the patients to improve their self-image, increase their personal security, socialize, develop their autonomy, and improve their talents. The interviewees also reported how objectives were highly tailored to the child and family’s needs and continuously adapted. For example:

*“The caregivers who came to our home helped my daughter socialize. They played together, pretending to be fashion designers, and did sewing activities. They played with magic sand, but most importantly, they talked a lot. They shared their difficulties, doubts, joys, and sorrows, and I can see how this helps my daughter to grow.”* (PP15)

*“The goals to be achieved with my daughter were built daily together with the caregiver. Sometimes, with specific activities like music therapy, because of her pathology, my daughter never went out. Hence, the goal was to have a person to help her realize her dream of becoming a singer.”* (PP13)

A second variable category, III/B (4/8), describes patients’ participation in the project, which occurred several times a week in various contexts. It is proposed in combination with other activities (e.g., physiotherapy, music therapy, play therapy, tasks, sewing, communication, psychoeducational support, and gardening). For example:

*“My little girl received two support activities from the project, music therapy, conducted at home, and homework assistance with an educator-psychologist who helped her with school, and they played together.”* (PP4)

*“Now they have suggested that we try hippotherapy with the doctor, along with music therapy and cognitive stimulation work. Altogether, the activities take place two to three times a week.”* (PP13)

The third typical category, III/C (6/8), highlights that the project activities were free for the families, conducted at home and elsewhere, and were chosen based on the needs and inclinations of the participants. Parents stressed the importance of the flexibility of the project, considering their family’s psychological needs and personalizing the project. For example:

*“We requested extra home activities to accommodate the family. Besides, my son was used to swimming, and I had to balance school, work, and his fatigue. But with the project’s flexibility, we were able to reconcile everything.”* (PP6)

*“The project has been helpful for my isolated family, which has no other assistance. The caregivers are a ray of light*, *an opportunity, and moral support during this difficult time. I hope it can continue; it moves me deeply.”* (PP13)

### Domain IV

Domain IV concerns the perceived barriers to the activities, with a typical category, IV/A (5/8), connected to the COVID-19 situation and the family’s fear of exposing the patient to dangerous situations. The lockdown measures were often seen as worsening the patients’ and their families’ psychological aspects and as interrupting or limiting many activities. For example, one mother said:

*“Having a daughter with leukemia during a global pandemic was bad luck because it drove us away from the project, which was helping the whole family to recover. However, when the incidence rates of contagion increased, we decided to stop the project to avoid the inevitable.”* (PP4)

On the other hand, the rare category IV/B (2/8) shows that, occasionally, the project was less effective in getting to know the patients and engaging them over time. This happened when there was a lack of personalization due to the healthcare professional not gaining a thorough initial knowledge of the child and their needs or missed communications with the hospital healthcare team. For example, a mother reported:

*“The effects were generally positive, but some practitioners had no connection with the hospital; they did not know my son’s situation well. This has led to some difficulties. But when there were activities that were a collaboration between the hospital and the home, my son was delighted and felt more comfortable.”* (PP4)

### Domain V

Domain V describes the project’s positive effects on the participating children and their families. This domain contains two general categories. Category V/A (8/8) describes the positive effects on the child in helping them to feel happy, quiet, and protected, sustaining their development, improving their socialization and autonomy, and receiving useful information about their physical condition. One mother reported on the positive effect of the project as follows:

*“My son had the opportunity to share his time of illness outside the home with the psychologist who knew how to manage his resistance, and I saw him gradually become happier and carefree.”* (PP4)

Category V/B (7/8) outlines the family support and facilitation of communication and collaboration with the healthcare professional and hospital. Often, mothers reported the importance of feeling part of a social support network, which counteracted their isolation and the family’s self-isolation. According to one mother:

*“The professionals were dedicated to implementing the interventions. The important thing is that they knew how to talk to us as parents to get to know our daughter better*.

*In my opinion, the activities have many benefits, more emotional than physical. These benefits also concerned the family members because they considered us an active part, which allowed us to recover and manage the time to work from home better. They provided moral support and fostered a bond with the professionals at the hospital.”* (PP06)

### Domain VI

Domain VI concerns the perceived feelings of patients associated with the activities. This domain contains a general category, VI/A (6/8), which highlights how participation in the activities induced positive emotions. For example:

*“For me, the caregiver is truly a kind, professional, and incredibly sensitive person. My son is very attached to her and feels very comfortable. Our participation in the project makes us feel good.”* (PP9)

*“A beautiful relationship has developed between our family and the caregivers. The project helps us tremendously. Moreover, the caregivers are lovely and always available, and we see our children happy. In short, participating has been very important for us.”* (PP13)

Another typical category (VI/B; 5/8) highlights that some patients felt tired, anxious, or angry towards certain people or activities related to the project. Some patients perceived the project activities as an additional commitment, requiring them to invest more energy in addition to that needed to manage their condition.

*“My son attends the activity in the pool, but understandably, sometimes he feels tired because he has to juggle many things*.

*It is also challenging for us, as family members, to balance school, work, and a series of other things, so we try to do our best. My son tells me he’s tired because he has to study and participate in activities, and he’s right.”* (PP6)

*“With one educator, my daughter experienced some discomfort; every time the activity with her ended, she would tell me that she wasn’t very happy. My daughter is very empathetic but couldn’t establish a good relationship with the facilitator*.

*Honestly, I also couldn’t form a connection with this professional, so perhaps it was a personal character issue with the caregiver. However, it went well for the others we have met.”* (PP13)

### Domain VII

Domain VII contains suggestions and requests to improve the project in the future, with a typical category, VII/A (6/8), mentioning ways to intensify and improve the activities. All the interviewees stressed that the project should continue and develop in the future and that it should do more to help the families cope with their child’s illness, especially in the initial phase of the disease when there is a lot of uncertainty and insecurity. For example, one mother said:

*“I would appreciate it if the caregivers went to the hospital at the beginning, maybe once or twice, when the child is still there, to have an initial introduction.”* (PP4)

Another way to expand the project’s reach is within the family and their relationship with the community. One mother said:

*“I hope that in the future, the children who participate in the project can be known from the start when they are at the hospital. This initial acquaintance with all the people involved in the project (doctors, psychologists, therapists, parents, and children) would allow for networking and enable the caregivers to build trust with the parents.”* (PP4)

*“Home care and outdoor care could be combined to promote socialization and include siblings. In these situations, even siblings suffer, so it would be good to promote activities to do together.”* (PP15)

### Domain VIII

During the interviews, the hospital was sometimes seen as a place of illness or a venue associated with stressful events and restrictions. This is reported in the variable category VIII/A (3/8), referring to the negative physical or emotional state associated with hospital-based therapies. For example, when a mother was asked what kind of relationship her child had developed with the psychologist who was seeing her at the hospital, she replied:

*“For my daughter, the idea of the hospital is always a drama because she still has nightmares about undergoing examinations, making her nervous. The thought of going to the hospital is filled with anxiety, even though she tries not to show it to me.”* (PP4)

Another mother stated:

*“The oncology department psychologist suggested that my child try to join in with the other hospital’s teens to socialize, talk about her illness, and perhaps make her understand that there is something else besides her illness. But my daughter won’t entertain going to the hospital, seeing all the things that happen there, the diseases and the problems.”* (PP8)

### The Special Case of Family PP14

As an example of the project’s flexibility and adaptability, we will discuss the case of family PP14. In this family, the young boy with leukemia showed steady improvement. When he reached the age of 18, he began attending the local university. However, during the same period, the mother was diagnosed with Alzheimer’s. The father describes the event as follows:

*“So, both my son’s situation and my wife’s occurred one day after the other, so basically this was a double knockout blow, a 1–2 boxing punch.”* (PP14)

The presence of two family members with chronic conditions and the ongoing COVID-19 situation placed a significant strain on the family and presented a series of complex and distressing challenges.

*“Well … then there’s my wife’s situation, which has complicated everything quite a lot … everything, obviously, because what my wife could normally do as a mother and a homemaker, unfortunately, she hasn’t been able to for a few years now, so what do we do? We have to take action … someone has to … has to do things for her, you know, the family, has to … has to live, that’s how it is.”* (PP14)

Despite the challenges, the project successfully adapted its goals and interventions to provide targeted support to the entire family. Additionally, it helped with practical matters, partially alleviating the father’s heavy workload and responsibilities.

*“So, the professional who comes to our house is amazing because besides talking with my son and me about all our family’s needs, she also takes care of some paperwork and anything else we need. We absolutely need someone like her*.

*She also supports my wife, to whom she dedicates quite a bit of time … the time she has available, so I’m really very happy indeed about the help that the project gives us*.

*When she gives us an appointment, we wait for her with open arms because, for us, it’s like having an encyclopedia that helps us with every aspect: 360° help. So, the benefit of the help is really significant, and it’s not just help for my son, it’s also help for my wife, and for me, because we see her as a reference point when we have a problem or a doubt.”* (PP14)

## Discussion

Overall, the project was well received by both families and children. The LbP objectives, which aimed to provide social participation opportunities through play, sport, and leisure, alongside psychosocial, emotional, and relational support, were fully recognized and appreciated by the respondents (refer to Domains III and V in Table 3). These findings align with the existing literature on the importance of parenting and social support programs to reduce stress and improve coping and family functioning (Gill et al., 2021; Yang et al., 2022). Parents reported improvements in their children’s functioning, their own, and the overall family dynamics (Domain V). They often wanted more activities, hours, and involvement from other family members, indicating that the project effectively addressed real family needs (Domain VII).

The perceived improvements in the child’s social-emotional functioning (Domain VI) are particularly relevant. Studies show that children’s and adolescents’ chronic illnesses are often associated with different forms of emotional distress, including symptoms of anxiety, depression, and post-traumatic stress (Compas et al., 2012). Less frequently investigated are problems of disruptive and oppositional behavior (Compas et al., 2012). The fact that the parents in the LbP project perceived improvements in the child’s emotional state shows how the interventions changed the child’s behavior in their own setting and their daily life.

We believe that underlying the project’s success is its high level of tailoring of the psychoeducational plans to the needs of the child and the families and that the activities were free of charge. This approach allowed young people and their families to participate in the planning and organization of support activities, combining psychosocial care and education in response to their individual needs (Cowen et al., 2010). Such active participation is important to children and families because the emphasis is on the young person and their family as capable of contributing to their own health, and not just as service users (Saffer et al., 2018). The interventions focused on getting to know the children and families in their own contexts, informing them about different activity options, giving opportunities to try things out, and making better-informed choices. The interview results show that when the project professionals overlooked this process, the parents noted this as a major defect (see Table 3, Domain IV).

The interview results also highlight the importance for the families and children to participate in activities that are run in locations connected to a sense of normalization; indeed, parents often mentioned the comfort of being at home (Domain VI). These findings align with the previous research of Jibb et al. (2021), showing that parents and children undergoing homecare have more opportunities to engage with friends, siblings, and each other while developing strong therapeutic relationships with their clinicians. This finding should also be interpreted in view of the fact that the COVID-19 pandemic was still ongoing when the project was run and meant that families saw the home as a safe place.

The findings related to doing things in a familiar or neutral context could better inform stakeholders on the importance of choosing the child’s home or other natural or public places as venues for psychosocial activities. Given that the main goal of chronic disease health management in children is to promote and support their best possible development in all areas and to prevent or reduce dysfunctions on behavioral, emotional, and social levels (Gill et al., 2021), this process should start with the choice of a venue that encourages positive development. In our analysis, this suggestion is reinforced by Domain VIII (“The Hospital as a Negative Place”). Indeed, the hospital was often mentioned as a place connected with stress and pain with interviewees repeatedly reporting that their child did not willingly attend the hospital. Therefore, any activities that could be performed elsewhere (e.g., psychotherapy, peer group meetings) should occur in another location whenever possible.

Another important finding associated with project personalization was that when the educators and healthcare professionals visited the children and families in their homes, they could dynamically adapt and consider activities based on a spur-of-the-moment assessment (Domain III). The need for flexibility is typical for those working with children with a medical condition at home (Capurso et al., 2021), showing that planning interventions must always be flexible and that the professionals’ interpersonal skills to read an ever-changing and fragile situation and adapt accordingly are essential. The main example of adaptation is that the LbP project initially aimed to involve young people and their families in social and communal activities outside their homes. However, due to the COVID-19 measures and concerns about ongoing contagion risks, project professionals had to change or adapt many of the activities to be done at home. Another example of the project’s ability to adapt is seen in the case of family PP14, as described at the end of the Results section above.

The findings, connected to the parents’ appreciation of the project and the critiques of things that did not work well, highlight the importance of conducting a family-based initial assessment and discussion. Such a process will gather knowledge of the specific needs and characteristics of the family and subsequently enable personalization of the types and modes of intervention delivery. However, in the context of childhood illness, greater intensity and complexity of interventions do not necessarily lead to better outcomes. For example, additional schedules to the daily agenda may create more burdens for parents, children, and families (Mitchell et al., 2021). A discussion with the family and the young person with a chronic illness allows for an assessment of the individual’s wishes and aptitudes while considering the family’s concerns or fears. Discussion also facilitates communication with the hospital-based healthcare team, which can positively affect patient compliance. Through dialogue with the family members, a professional can consider aspects that might otherwise be easily overlooked, such as logistics (e.g., transport, equipment), physical strains connected to the participation in a specific activity, or interference with other children or family occupations.

## Conclusion

From a systemic perspective, interventions with children with a medical condition and their families often have effects that extend beyond the immediate family unit. Such interventions can lead to positive changes at various levels, improving the overall health and well-being of the entire family by altering their perception of control and interactions with the external environment.

Implementation of evidence-based interventions require the involvement of a caring professional who can establish a compassionate relationship with the families and children (Cousineau et al., 2019). From the family’s perspective, listening is essential for patient-centered care and the customization of activities. It is also a fundamental requirement for a strong therapeutic alliance as it enables young patients and their families to actively participate in their treatment and adopt a collaborative approach toward recovery or improving their quality of life (Epstein & Street, 2011).

More healthcare activities are increasingly being transferred to the home (National Research Council, 2011). This study supports such the existing literature by showing that it is not only medical activities that need to be transferred. Various psychoeducational services must be provided to the patient and their family to promote resilience and development at home. This often requires new professional skills, effective listening, and the ability to coordinate among all professionals involved in home healthcare practice.

### Study Limitations

The project evaluation was limited to a small sample of participants, all associated with a single pediatric care center in Palermo. Further, the study was constrained by the availability and willingness of participants to share their experiences and perspectives. We are unaware of why some families declined to consent to participate in the evaluation interview. Nevertheless, this qualitative study provides a wealth of data derived from the perspectives of participants involved in a specific program that aligns with the objectives of this research paper.

Finally, self-report testimonies and all the responses should be understood as the perceptions and viewpoints of the individuals interviewed and, thus, are subject to potential bias. Several measures were implemented to minimize interview bias. First, a semi-structured interview protocol was used, ensuring all participants were questioned about the same main topics. Additionally, the interviews were conducted by a trained interviewer, who followed a jointly constructed discourse with the participants. The researcher was blinded to the identity of the patients and interviewees, and all participants were informed of that. Furthermore, general statements made by the interviewees were validated by requesting specific examples to enhance the reliability of the information collected. This approach also facilitated the subsequent coding of the text.

Despite these limitations, the data provide valuable insights. The findings validate the project (with 100% of the interviewees reaching a consensus on the positive project outcome) while depicting specific aspects of how the project activities were experienced within each family.

### Implications for Further Research and Development of the Intervention

Training of home-based professionals is crucial for these types of projects to be successful. Different content can be delivered in such training, such as developing listening and empathic abilities. These skills are particularly important, as they greatly contribute to the successful outcome of the project. One example of this type of training could be the Thomas Gordon Effectiveness Program (Gordon & Burch, 2003) or any other established counseling course (Pieterse et al., 2009), which can be delivered in the form of intensive workshops to meet the needs of working professionals. Another type of training that would benefit similar projects relates to the BES (refer to Supplementary File 1). Since the interventions are based on different BES, professionals must become aware of the meaning, significance, and psychological dynamics that define the particular BES they are working on with each intervention. A workshop structure would enable professionals to actively develop ideas on various activities and learn how to connect them with each BES.

The component of involving families in their own healthcare can be further developed. For instance, families could gradually assume responsibility for organizing specific community events, while volunteer associations, deeply rooted in the community, could scale back their involvement and provide logistical and administrative support instead (e.g., obtaining public permits, arranging transport, securing reception facilities, renting public spaces). This would make it easier to support the activities in the long term. Continuous support for communication between the family, the hospital, and its practitioners is paramount. One possible solution could be to appoint a liaison figure who collects and shares relevant information between the family and the hospital (and vice versa).

Another area that would benefit for further study is the role of a designated project person responsible for documenting the project to prevent the scattering of knowledge and important information. This might involve creating a manual that would make it easier to train new professionals and transfer/adapt the project to different contexts.

Another potential area for investigation could be to conduct a comprehensive analysis of the gender perspective. Given that that seven out of eight participants in our study were women, further investigation could explore how the role of mothers as caregivers can be enhanced and supported within the family and the specific sociocultural context in which they reside (Giorgio, 2015).

Finally, most services have a monetary cost. Future research should focus on implementing a wider range of home-based services for child and family health. Such research should consider the cost and effectiveness of these services. In countries like Italy, with a universal healthcare system, extending these services to all citizens is challenging. In countries with private healthcare, the challenge is recognizing home-based psychoeducation services as a fundamental part of patient and family care.

## Additional File

The additional file for this article can be found as follows:

10.5334/cie.65.s1 Supplementary File 1. List of the Basic Experiences of the Self (BES; Rispoli, 2008).
