# Supplementary material for: Tailored Psychoeducational Home Interventions for Children with a Chronic Illness: Families’ Experiences
Source: Contin Educ. 2024 Jan 5;5(1):1–21. doi: 10.5334/cie.100 (PMC11104378; doi:10.5334/cie.100)
Supplement: Supplementary File 1. — List of the Basic Experiences of the Self (BES; Rispoli, 2008). [file cie-5-1-100-s1.pdf]

Supplementary File 1: The Basic Experiences of the Self (BES; Rispoli, 2008)

Being Held
Being Held, Being Contained (Being Stopped)
Protection

Being Picked Up
Being Picked Up
Being Carried

Letting Go
Letting Go (Daydreaming)
Just Being
Abandoning ourselves to….

Contact
Contact (Fusion)
Being Nourished (Absorbing)

Active Contact
Taking (Asking, Seducing, Drawing Others to us)
Giving (Hugging)
Giving to Ourselves (Consoling Ourselves, Finding Balance)

Love
Being Loved (Belonging, Being Carried inside the other person)
Loving (Carrying the other person inside us)
Positive Continuity (Memories)

Sharing
Opening Up
Pleasure in Others
Pleasing Others (Showing Ourselves)

Tenderness
Needing Others
Tenderness
Fragility (Softness)

Receiving Attention
Being Seen
Being Listened to
Being Understood
Being Helped
Being Encouraged

Vitality
Joy (Excitement, Bursts of Energy)
Vitality (Energy, Impetus)
Playing (Running, Throwing Ourselves into…)

Well-Being
Well-Being (Harmony, Wholeness)
Pleasure (Vagotonia, Mobilizing towards the Lower Body)

Sensations
Perceiving
Sensing (Sensations)
Amazement

Control
Losing Control (Explosions)
Loosening Control (Head Bent, Collapses)
Soft Attention

Strength
Original Strength (Detachment, Space)
Soft Strength
Calm Strength
Open Strength (Letting Out)
Changing Others (Moving)

Aggressiveness
Affectionate Aggressiveness
Aggression

Authority
Presence (Determination)
Authority (Standing, Confidence, Knowing Our Own Value)

Assertiveness
Assertiveness
Making Choices
Making Plans

Negativity
Anger
Hatred

Autonomy
Opposition
Separation
